# Out-of-pocket payments in the context of a free maternal health care policy in Burkina Faso: a national cross-sectional survey

**DOI:** 10.1186/s13561-019-0228-8

**Published:** 2019-03-27

**Authors:** Ivlabèhiré Bertrand Meda, Adama Baguiya, Valéry Ridde, Henri Gautier Ouédraogo, Alexandre Dumont, Seni Kouanda

**Affiliations:** 1 Département Biomédical et Santé Publique, Institut de Recherche en Sciences de la Santé (IRSS/CNRST), 03 BP 7192, Ouagadougou, Burkina Faso; 2 École de Santé Publique de l’Université de Montréal (ESPUM), Montréal, Canada; 3 Institut de recherche en Santé Publique de l’Université de Montréal (IRSPUM), Montréal, Canada; 4 Institut Africain de Santé Publique (IASP), Ouagadougou, Burkina Faso; 5 IRD (French Institute for Research on Sustainable Development), CEPED (IRD-Université Paris Descartes), Universités Paris Sorbonne Cités, ERL INSERM SAGESUD, Paris, France

**Keywords:** Free care policy, Maternal care, Direct expenses, Out-of-pocket payment, Sub-Saharan

## Abstract

**Background:**

In April 2016, Burkina Faso introduced a free health care policy for women. Instead of reimbursing health facilities, as many sub-Saharan countries do, the government paid them prospectively for covered services to avoid reimbursement delays, which are cited as a reason for the persistence of out-of-pocket (OOP) payments. This study aimed to (i) estimate the direct expenditures of deliveries and covered obstetric care, (ii) determine the OOP payments, and (iii) identify the patient and health facility characteristics associated with OOP payments.

**Methods:**

A national cross-sectional study was conducted in September and October 2016 in 395 randomly selected health facilities. A structured questionnaire was administered to women (*n* = 593) who had delivered or received obstetric care on the day of the survey. The direct health expenditures included fees for consultations, prescriptions, paraclinical examinations, hospitalization and ambulance transport. A two-part model with robust variances was performed to identify the factors associated with OOP payments.

**Results:**

A total of 587 women were included in the analysis. The median direct health expenses were US$5.38 [interquartile range (IQR):4.35–6.65], US$24.72 [IQR:16.57–46.09] and US$136.39 [IQR: 108.36–161.42] for normal delivery, dystocia and cesarean section, respectively. Nearly one-third (29.6%, *n* = 174) of the women reported having paid for their care. OOP payments ranged from US$0.08 to US$98.67, with a median of US$1.77 [IQR:0.83–7.08]). Overall, 17.5% (*n* = 103) of the women had purchased drugs at private pharmacies, and 11.4% (*n* = 67) had purchased cleaning products for a room or equipment. OOP payments were more frequent with age, for emergency obstetric care and among women who work. The women’s health region of origin was also significantly associated with OOP payments. For those who made OOP payments, the amounts paid decreased with age but were higher in urban areas, in hospitals, and among the most educated women. The amounts paid were lower among students and were associated with health region.

**Conclusion:**

The policy is effective for financial protection. However, improvements in the management and supply system of health facilities’ pharmacies could further reduce OOP payments in the context of the free health care policy in Burkina Faso.

## Background

Since the Bamako Initiative in 1987, many countries in Sub-Saharan Africa have adopted out-of-pocket (OOP) payments at the point of service as a method for financing healthcare. However, there is currently a broad consensus that OOP payment for services is a barrier to health care access, especially for the poorest. It has also been found to expose households to a risk of catastrophic expenditure and thus to impoverishment [1, 2].

For those reasons, in the late 1990s and early 2000s, several countries in Sub-Saharan Africa introduced public policies that eliminated or reduced fees for specific social groups or specific types of care [3]. These policies were also intended to speed up the achievement of some of the Millennium Development Goals (MDGs), including maternal, newborn and child health related goals. These policies vary by country in terms of the services covered, the social groups benefited and the cost mitigation level [4].

However, the abolition or reduction of user fees deprives health facilities of revenue that enables them to cover operational expenses. Generally, the state compensates for this loss of revenue, and payment methods vary by country. Several countries reimburse a lump sum per service delivered, and the rate may depend on the level of care or facility ownership. However, many authors have noted that the methods used to calculate these rates are unclear [4–6]. For example, in May 2010, Burkina Faso shifted from the case-based reimbursement method to the fee-for-service method (reimbursement of actual expenses) as part of its subsidy policy for deliveries and emergency obstetric and newborn care (EmONC) because it was found that the fixed rate exceeded the actual expenses [5]. In contrast, in Niger, the free care policy for children under five under-reimbursed health facilities [5]. Knowing the actual expenses of medical care and services can prevent incorrect reimbursement to health facilities and also allow the correct estimation of the financial sustainability of these policies. Further, knowledge of actual health care expenses can allow the escalation of these expenses to be tracked and effective cost containment measures to be taken if necessary. Several authors have called for the need to calculate the real expenses of services covered by fee abolition policies [6, 7].

Furthermore, several studies have demonstrated that OOP payments persist within the context of free care policies [8–14]. This persistence of OOP payments is explained by factors including the unavailability of drugs in health facilities, the loss of health facility revenues and reimbursement delays [9]. However, these studies [8, 9, 12, 15] are often limited in geographical scope, for example, to one health district. This approach does not allow comparisons across districts regarding, for example, the persistence of OOP payments when the effects of the free care policies are later assessed. In addition, few studies have explored the patient and health facility characteristics that could be associated with the persistence of OOP payments in the context of free care policies [13, 16].

In April 2016, Burkina Faso introduced a free care policy for women and children under five. This free care policy was implemented after ten years of a subsidy policy that covered 80% of direct medical expenses for deliveries and EmONC [17]. However, instead of reimbursing health facilities, as many sub-Saharan countries do [4], Burkina Faso paid them prospectively for covered services to avoid reimbursement delays, which have been found to promote the persistence of OOP payments. Thus, we conducted a national study six months after the introduction of the free care policy with the aims of (i) calculating the direct health care expenses of delivery and covered obstetric care, (ii) determining the proportion of women who make OOP payments and the amounts of those payments, and (iii) identifying the patient and health facility characteristics associated with OOP payments.

The study was carried out with regard to two perspectives. From the government’s perspective, it is important to estimate the costs of the free care policy if the state should bear all direct health care expenses of the covered services, and a better knowledge of the actual average expenses incurred for each covered service is necessary for this estimation. From the patient and his household’s perspective, it is useful to know the amount of direct health expenditures that remain chargeable to them.

## Methods

### Study setting

Burkina Faso is a low-income country where the public health care system is organized on a pyramidal basis with three levels. The first level is the health district, which comprises basic health centers, called “Centre de Santé et de Promotion Sociale (CSPS)”, medical centers (MCs) and district hospitals. In 2016, there were 72 health districts with 1760 CSPSs, 52 MCs and 47 functioning district hospitals. In 2016, the second level comprised eight regional hospitals, and the third level comprised five University Teaching Hospitals (UTH), including an exclusively pediatric center that does not perform deliveries [18].

CSPSs provide normal deliveries that are performed by midwives, auxiliary midwives, nurses or auxiliary nurses. CSPSs with midwives also perform deliveries involving dystocia, postpartum hemorrhage management and intrauterine manual vacuum aspiration. MCs have general practitioners in addition to CSPS staff, but they are not equipped for surgery.

Obstetric emergencies (cesarean section, ectopic pregnancy, eclampsia crises, etc.) are managed at district hospital, regional hospital and UTH. Cesarean section is performed by obstetrician-gynecologists, general practitioners trained in emergency surgery and nurses with three years’ training in surgery who are called “Attachés de santé en chirurgie” in Burkina Faso.

### Free care policy for women

In March 2016, a decree was adopted by the government of Burkina Faso establishing a free care policy for women. It was first implemented at the CSPSs, MCs and district hospitals in three regions (Central, Hauts-Bassins and Sahel) on April 1, 2016. On May 1, 2016, it was extended to the regional hospital in Sahel and UTH in the Central and Hauts-Bassins regions before being implemented all over the country starting on June 1, 2016.

The benefit package includes antenatal care, normal deliveries and EmONC, curative care during pregnancy and up to 42 days after delivery, treatment of obstetric fistulas, screening and in situ treatment of precancerous cervical lesions for women between 25 and 55 years old and clinical screening for breast cancer starting at age 15. Antenatal care incorporates the prevention of anemia and malaria, urine testing for albumin, blood grouping, hemoglobin electrophoresis and screening for syphilis. EmONC includes dystocia, cesarean sections, laparotomy for uterine rupture or ectopic pregnancy, pre-eclampsia or eclampsia, post-abortion care and newborn intensive care.

The covered expenses for all targeted services under the free care policy include fees for consultation or surgery, prescriptions fees, paraclinical examinations (laboratory tests and medical imaging), hospitalization expenses and the expenses of ambulance transportation between health facilities. Eligible women should not be paying for these components. In fact, the state acts as a third-party payer for health facilities.

The free care policy is fully funded by the state budget. Health facilities are paid according to a fee-for-service method with scheduled fees. However, payments are made prospectively rather than retrospectively, as it is typically the case for fee-for-service payments. Through this approach, the government avoids reimbursement delays, which is considered the main barrier to the success of cost-reduction policies in Sub-Saharan Africa. In practice, the funds are pre-deposited quarterly into hospital and district accounts based on centrally determined allocation keys based on the services that each health facility is expected to provide. This estimation considers the historical utilization over the six last months. Health facilities use this money and produce monthly reports on the services provided and their expenses to the Ministry of Health. At the beginning of the next quarter, funds are transferred again in consideration of the bank account balance.

The monthly activities and financial reports of the facilities are checked at the district, regional and central levels. A sample of health facilities is surveyed monthly by four international nongovernmental organizations (NGOs) selected by the Ministry of Health. The selection of health facilities is mainly determined by the suspect nature of their reports. This survey consists of the following: (i) checking the consistency between the data transmitted in reports and those in the health facility’s registers, (ii) conducting exit interviews of a random sample of patients to measure their satisfaction with the care they received and to ensure that the drugs they received correspond with those listed in registers, and (iii) conducting household surveys to ensure that the beneficiaries reported in the registries truly exist. Cases of fraud are submitted to the administrative authorities for sanctions according to the procedures established within public service.

### Study design

This was a national cross-sectional study conducted in public health facilities from September to October 2016. It was conducted by “Institut de Recherche en Sciences de la Santé” during the annual needs assessment in reproductive health funded by the United Nations Population Fund (UNFPA). A structured questionnaire was used to collect data.

### Study population and sampling

The study population comprised women who had delivered or received emergency obstetric care at a public health facility during the study period.

We used a multistage stratified sampling with facility types (hospitals, MCs and CSPS) as the strata. All of the hospitals and MCs were included in the sample because they were few in number. We then selected, by simple random sampling, one-fifth of the CSPSs in each region. At each CSPS, MC and district hospital, we chose one normal delivery without episiotomy, one with episiotomy and one case for each type of emergency obstetric care (EmOC). EmOC includes dystocia with and without episiotomy, postpartum hemorrhage, intrauterine manual vacuum aspiration, cesarean section and eclampsia. The last two services are provided only at hospitals. At the regional and university hospitals, five and ten cases of each type of care cited above were randomly selected, respectively. The services selected were those covered by the free care policy. The number of cases per type of service was higher for the regional and university hospitals to ensure significant total numbers for the statistical calculations given the relatively low number of these types of health facilities.

Women who received care at the selected health facilities during the interviewer’s visits were included in the selection of the sample. The length of stay at health facilities varied from a few hours to several days according to the type of service. The sample included only women whose care for the episode had been completed and who were still present at the health facility. This limitation was to ensure that all medical expenses for the episode were included.

The sample size of women was determined by the following formula [19]:

$$ \mathrm{n}\kern0.5em =\kern0.5em \frac{1{.96}^2\kern0.5em \times \kern0.5em \mathrm{p}\kern0.5em \times \kern0.5em \left(1-\mathrm{p}\right)\kern0.5em \times \kern0.5em \mathrm{DEFF}}{{\mathrm{d}}^2} $$

where the percentage of births attended by skilled health personnel in 2016 *p* = 0.809, the level of absolute precision d = 0.05 and the estimated design effect DEFF = 2. We obtained a minimal sample of 475. This sample size was to ensure that the estimated expenses were representative of skilled birth attendance.

### Measure of outcomes

There were two outcome variables: OOP payments and direct health care expenses. OOP payments represented the total expenses paid by each patient for the following components: fees for consultation and/or surgical intervention, prescription fees, paraclinical examinations, hospitalization and ambulance transport between health care facilities. These expenditures could be paid inside or outside the health care facility; in particular, outside payments were made for drugs and paraclinical examinations that were not available at the facility. Unofficial payments to health professionals for drugs or care were included in the calculations. The data sources were payment receipts and patient reports. Patient reports were double-checked with the receipt except for unofficial payments (payments to health professionals, for cleaning products, etc.) that had no receipt. However, when unofficial payment practices were found to exist in a health facility, they were common and we confirmed them with other patients. Only expenses eligible for free care were included. Consequently, expenditures for food and transportation (except by ambulance) were not included in the OOP payments.

We defined the direct health care expenses of a service as the expenses covered by the free care policy. This definition includes expenses charged to the Ministry of Health by the health facility and the OOP expenses borne by the patient. The free care policy sheets that summarize the services provided for each patient and their expenses were additional data sources used. This information was cross-checked with medical prescriptions and reports obtained from the patients.

The expenditures were calculated in local currency (XOF) and then converted into US dollars using the average exchange rate for 2016 (US$1 = XOF592.912968).

### Independent variables

In the study of factors associated with OOP payments, the independent variables included patient sociodemographic characteristics (age, education level, marital status, place of residence, parity at admission) and health system-related characteristics (health region, type of health facility, type of service and the service provider’s qualifications). Sociodemographic characteristics were collected from the patients, and health system-related characteristics were obtained from health professionals and by checking the registries.

Age was collected as a continuous variable and categorized into 5-year intervals. Parity was categorized into three groups: nulliparous, multiparous (1–4 deliveries) and grand multiparous (at least 5 deliveries). Health facilities were also grouped into three categories (CSPS**,** MCs and hospitals).

The level of education was divided into three categories: none, primary and secondary or higher level. Marital status included two categories, married and not married, whereas the service provider qualifications comprised five categories: physician, midwife, nurse, auxiliary midwife and surgeon’s assistant. The place of residence was divided into two categories, rural and urban, according to the national classification of the municipalities.

### Data collection

Data were collected from September to October 2016 by physicians and medical students. In total, there were sixty-six (66) data collectors, organized into 18 teams. Each team was supervised by a team leader. There were an average of six data collectors per region. They received a two-day training and conducted a pilot test. Each team leader was responsible for ensuring that all the forms were completed. In addition, we conducted two five-day supervisions with three other teams of two supervisors during data collection to ensure that the survey was properly conducted and that data quality was maintained.

The types and quantities of drugs and consumables used and paraclinical examinations performed were reported in the questionnaire. The prices of drugs and consumables were those charged by the health facility pharmacy or those reported on the prescriptions in cases where patients had paid at private pharmacies. Consultation fees, paraclinical examinations and hospitalization prices were specific to health facilities and were specified on health tariff policy sheets or in reports from the relevant service providers (the laboratory, for example).

In cases of discrepancy between the expenses reported on the free care sheets and the prices obtained from medical prescriptions or examination reports, we checked with health professionals.

### Data processing and analysis

We performed a double data entry with two trained and supervised data entry clerks using Epi Data. The data were then exported to Stata version 15.1 (Stata Corporation, Texas, USA) for quality check and analysis.

The analysis was conducted in two phases. First, we determined the direct health expenditures for normal delivery and each EmOC. For this purpose, descriptive statistics were used to describe the sample. Then, direct health care expenses were standardized by type of service, and observations with standardized values above 3.29 were deemed extreme [20]. Five such cases were noted, including four deliveries that required the administration of anti-D immunoglobulins and one case of postpartum hemorrhage. The four deliveries were excluded from the analysis because the use of anti-D immunoglobulins is a specific service, and too few patients received this service to form a separate group. Two other cases (cesarean section and dystocia) that did not meet the extreme values criteria but also required anti-D immunoglobulin were also excluded from the analysis. Then, we computed the mean and median of direct expenses for each service type at the national level and by type of health facility. We also calculated the median and mean of OOP payments separately for normal delivery and EmOC.

In the second phase, we investigated the factors associated with OOP payments. We used crosstabulation to ensure that there was no systematic relationship between certain independent variables. Women who made OOP payments were then compared according to their characteristics and the health facility characteristics using Pearson’s chi-squared test.

For the analysis of health expenditures data involving only nonnegative values, several authors recommended a two-part model approach [16, 21–23]. In this approach, the probability of OOP payment was first modeled, and then the amount of payment for those who paid was modeled. In general, the first part uses a probit or logit regression [21, 23]. We chose a logit regression because the interpretation of its results is quite straightforward. For the second part, the Box-Cox test (*p* = 0.958 for λ = 0) showed that the data are fit for use of the generalized linear model (GLM) with a link log. For the choice of family distribution, the authors recommended mainly Gamma or Inverse Gaussian family for health expenditures that exhibited skewness [21, 23]. Upon using the modified Park test, the result (*p* = 0.6167 for δ = 2) showed that the data are gamma distributed. Thus, the first part model may be conceptualized as follows:

$$ \mathrm{In}\kern0.5em \left[\frac{\mathrm{Prob}\left(\mathrm{y}>\left.0\right|\mathrm{x}\right)}{1\hbox{-} \mathrm{Prob}\left(\mathrm{y}>\left.0\right|\mathrm{x}\right)}\right]\kern0.5em =\kern0.5em \upalpha \kern0.5em +\kern0.5em \sum {\upbeta}_{\mathrm{i}}\kern0.2em {\mathrm{x}}_{\mathrm{i}} $$

where y represents the OOP payments, α is the constant term, x_i_ represents a set of independent variables and β_i_ are the estimated coefficients for these variables. *Prob*(*y* > 0| *x*) represents the probability that a patient will experience an OOP payment for normally free of charge service.

For the second part, the equation is the following:

$$ \mathrm{In}\kern0.5em \left[\mathrm{E}\left(\left.\mathrm{y}\right|\mathrm{x}\right)\right]\kern0.5em =\kern0.5em \upalpha \kern0.5em +\kern0.5em \sum {\upbeta}_{\mathrm{i}}\kern0.2em {\mathrm{x}}_{\mathrm{i}} $$

where E(y) represents the expected value of OOP expenses. The other notations share similar definitions as those in the first part of the model.

The two parts may be analyzed separately or together. However, analyzing the two parts together allows for prediction of the OOP expenses based simultaneously on the two models. We opted for the combined analysis but took advantage of the broad range of tools available in logistic regression and GLM to check each model individually. Observations that did not fit the models were checked and removed. For the multiple regression, we regrouped the different services into two categories, normal deliveries and EmOC, because certain EmOC services were exclusively offered in hospitals. We excluded the provider qualifications variable of the multiple regression analysis because the type of service performed depended on the provider’s qualifications. We also standardized the outcome variable OOP expenses for the second part of the analysis and excluded standardized values above 3.29 (two observations) from the analysis. The analysis was computed using the Stata command “twopm”. The statistical threshold was set at 0.05 for all statistical tests. Standard Errors were adjusted for the clustering of data at the district level. The parity variable was excluded from the final model because it did not significantly contribute to the model. Finally, we used the margins command to predict the amount of OOP payments for certain variables.

### Ethical considerations

This study was part of the 2016 reproductive health needs assessment and was approved by the Health Research Ethics Committee of Burkina Faso. The patients gave informed consent. In addition, survey data confidentiality was ensured by the anonymity of the collection tools.

## Results

### General characteristics of the sample

A total of 593 women were surveyed, and 587 were analyzed. The women were surveyed from 299 public health facilities, including 228 CSPSs, 21 MCs, 39 district hospitals, 8 regional hospitals and 3 university hospitals. 67% of these women had no education and 19.2% had a primary school education level. Women under 20 years and those aged 20–24, 25–29 and 30–34 years accounted for 27.8%, 26.2%, 20.6% and 13.1% of the sample, respectively. Most of the surveyed women were married (94.7%) and were housewives (84.7%), and three-quarters (76%) lived in rural areas. Multiparous women represented 53.7% of the sample, whereas nulliparous women represented 37.1%. Midwives (47.4%) and auxiliary midwives (29%) provided most of the services. Physicians, assistant surgeons and nurses provided only 7.8%, 2.7% and 13.1% of the services, respectively.

### Direct health care expenses of deliveries and obstetric emergencies

Table 1 shows the mean and median health care expenses for deliveries and obstetric emergencies, calculated at the national level and by health facility type. Expenditures increased according to the complexity of the services provided and the level of care. For example, at the national level, the median expenditures for normal delivery, dystocia management and cesarean section were US$5.38 (XOF3,190), US$24.72 (XOF14,655) and US$136.39 (XOF80,870), respectively.

**Table 1:** Direct healthcare expenses mean (standard deviation) and median (interquartile range) in US$ by type of service at the national level and type of facility in Burkina Faso in 2016

	National	Health center	Medical center	Hospital
Normal delivery				
n	246	209	18	19
Mean (SD)	6.12 (±2.98)	5.58 (±2.04)	7.74 (±3.84)	10.52 (±5.71)
Median (IQR)	5.38 (4.35–6.65)	5.09 (4.22–6.31)	6.38 (5.30–8.96)	7.56 (5.70–13.38)
Normal delivery + episiotomy				
n	139	112	16	11
Mean (SD)	12.04 (±4.68)	11.01 (±3.27)	13.64 (±6.75)	20.28 (±4.99)
Median (IQR)	10.66 (8.97–14.33)	10.04 (8.80–12.87)	10.85 (9.68–15.85)	19.89 (16.49–21.15)
Dystocia				
n	59	12	3	44
Mean (SD)	31.61 (±19.77)	12.88 (±5.37)	NC	37.26 (±19.45)
Median (IQR)	24.72 (16.57–46.09)	11.22 (8.99–16.70)		34.36 (21.24–51.99)
Dystocia + episiotomy				
n	47	12	1	34
Mean (SD)	33.53 (±19.84)	14.63 (±3.77)	NC	40.54 (±18.96)
Median (IQR)	31.04 (17.29–47.54)	13.71 (12.93–17.26)		36.51 (26.40–50.76)
Postpartum hemorrhage				
n	21	1	2	18
Mean (SD)	56.68 (±41.55)	NC	NC	63.88 (±40.58)
Median (IQR)	58.35 (29.56–64.56)			59.66 (39.13–64.71)
Eclampsia				
n	7	NA	NA	7
Mean (SD)	49.33 (±19.87)			49.33 (±19.87)
Median (IQR)	42.73 (32.48–72.99)			42.73 (32.48–72.99)
Post-abortion care		NA	NA	
n	19			19
Mean (SD)	45.89 (±33.99)			45.89 (±33.99)
Median (IQR)	32.10 (22.31–54.61)			32.10 (22.31–54.61)
Caesarean section		NA	NA	
n	49			49
Mean (SD)	141.84 (±42.96)			141.84 (±42.96)
Median (IQR)	136.39 (108.36–161.42)			136.39 (108.36–161.42)

*NC=* Not calculated (small number of cases), *NA* = Not applicable (the service is not available at this level of care)

Average exchange rate in 2016: US$1 = XOF592.912968

**Fig. 1:**
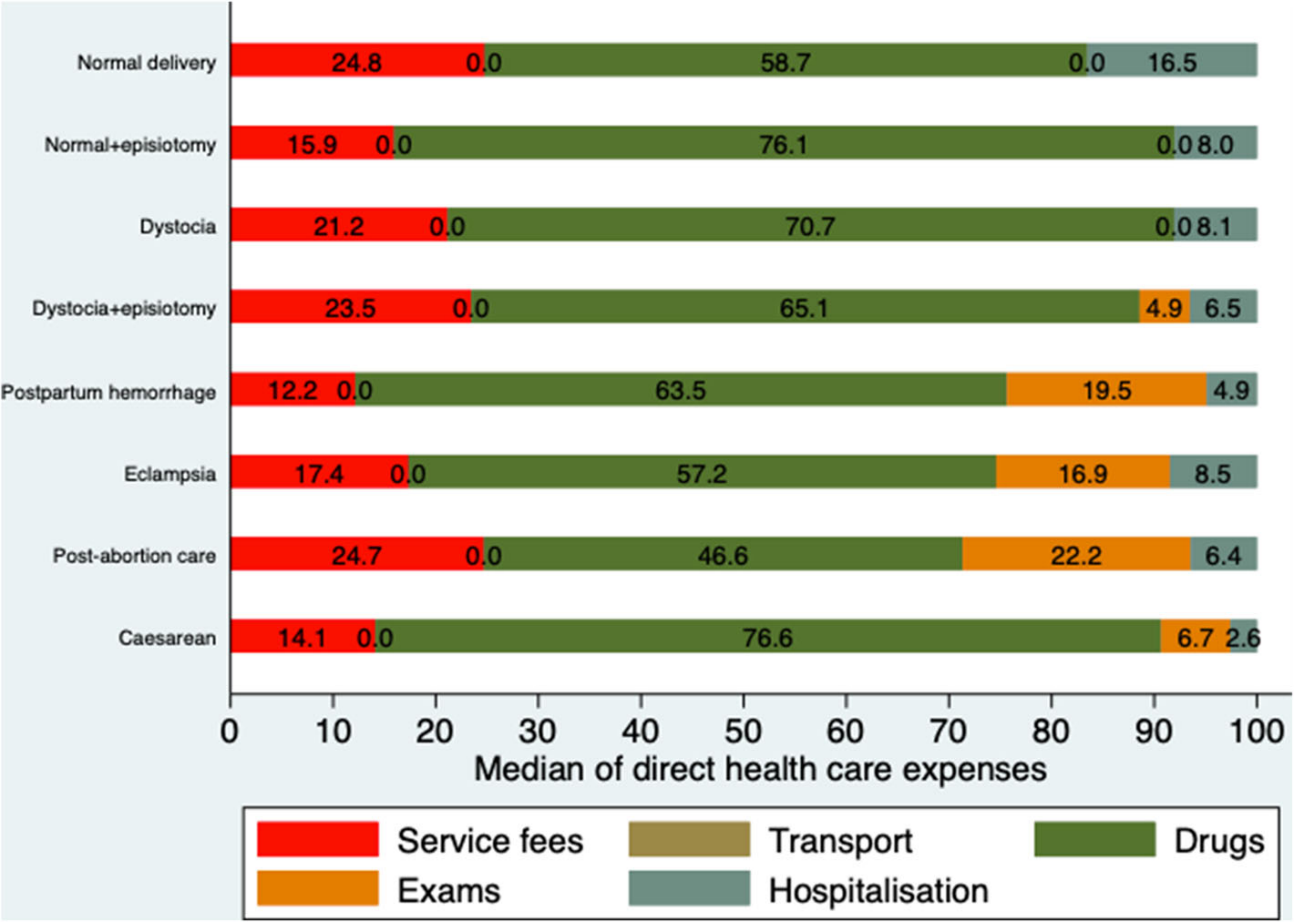
Shares (in percentages) of the service components of median direct health care expenses by service type in Burkina Faso in 2016

Figure 1 presents the shares of the service components of median health care expenses for each service type. Across all service types, drugs and consumables accounted for the largest share of median health care expenses, ranging from 46.6% for post-abortion care to 76.6% for cesarean section.

### OOP payments for delivery and emergency obstetric care

**Fig. 2:**
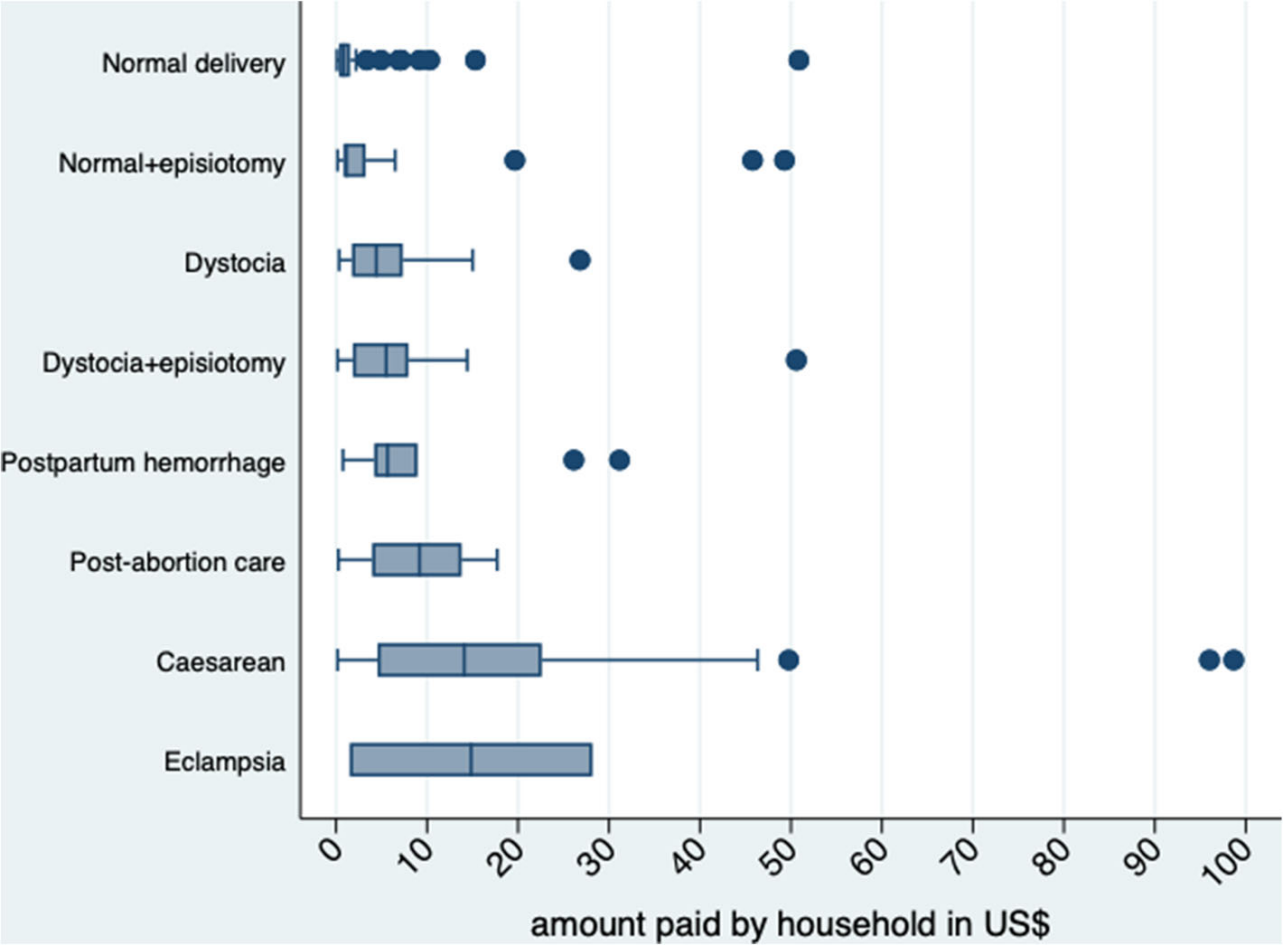
Distribution of amount paid by household according to the type of service

Overall, 29.6% (*n* = 174) of women paid direct medical expenses. In absolute terms, the amounts paid by these women ranged from US$0.08 to US$98.67 with a median of US$1.77 [interquartile range (IQR): 0.83–7.08] for all services combined. For normal delivery, the amount paid by women ranged from US$0.08 to US$15.31 with a median of 0.84 [IQR: 0.34–1.43]. For normal delivery with episiotomy, the amount paid ranged from US$0.17 to US$19.65 with a median of US$1.05 [IQR: 0.80–2.02]. For dystocia, the amount paid ranged from US$0.34 to US$26.82 with a median of US$4.43 [IQR: 1.77–7.31]. For dystocia with episiotomy, the amount paid ranged from US$0.17 to US$14.42 with a median of US$5.42 [IQR: 1.77–7.46]. For a C-section, the amount paid ranged from US$0.17 to US$98.67 with a median of US$13.78 [IQR: 3.96–21.67]. Figure 2 shows the distribution of the amount’s women paid for each service type.

Table 2 (a and b) presents the OOP payments made by the women for normal deliveries and for obstetric emergencies by each expense component. Most OOP payments were made for cleaning products (14.3%) in case of normal deliveries and for drugs at private pharmacies (34.2%) in case of obstetric emergencies because they were either out of stock or not sold at the health facility pharmacy. Drugs not sold at the pharmacy were essentially specialty drugs. However, irrespective of the service category (normal deliveries or EmOC), the total OOP expenses and the drug expenses exhibited similar coefficients of variation (CV), and drug expenses were higher than the other service components. The CV for drug expenses and total OOP expenses were 128% and 163% in the case of normal deliveries, 146% and 147% in the case of EmOC, respectively.

**Tables 2:** Out-of-pocket payments (in US$) under free care policy in Burkina Faso in 2016

	n	% women	Mean (SD)	Median (IQR)	Min	Max
a) Normal deliveries (*N* = 385)						
Consultation fees	0	0	–	–	–	–
Drugs expenses – private pharmacies	34	8.8	2.99 (3.82)	1.43 (0.51–3.88)	0.25	15.31
Cleaning products	55	14.3	1.04 (1.16)	0.51 (0.17–1.43)	0.08	5.06
Paraclinical exams	0	0	–	–	–	–
Hospitalization fees	13	3.4	0.84 (0)	0.84 (0.84–0.84)	0.84	0.84
Transport	0	0	–	–	–	–
Drugs expenses – care provider	2	0.5	0.93 (0.12)	0.93 (0.84–1.01)	0.84	1.01
Total expenses	89	23.1	1.93 (3.14)	0.84 (0.42–1.69)	0.08	19.65
b) Obstetrics emergencies (*N* = 202)						
Consultation fees	2	1.0	1.77 (1.07)	1.77 (1.01–2.53)	1.01	2.53
Drugs expenses – private pharmacies	69	34.2	11.97 (17.42)	5.90 (3.88–14.67)	0.25	98.67
Cleaning products	12	5.9	1.76 (2.08)	0.76 (0.38–2.53)	0.17	6.41
Paraclinical exams	5	2.5	12.48 (8.21)	10.12 (6.75–13.49)	5.90	26.14
Hospitalization fees	2	1.0	1.94 (2.03)	1.94 (0.51–3.37)	0.51	3.37
Transport	1	0.5	3.37	3.37	3.37	3.37
Drugs expenses – care provider	8	4.0	5.45 (6.51)	2.61 (0.84–9.74)	0.34	16.87
Total expenses	85	42.1	11.34 (16.68)	6.04 (3.04–13.79)	0.17	98.67

*SD* = Standard deviation, *IQR* = Interquartile range, *%* = proportion of women among those who experienced obstetric emergencies

Obstetrics emergencies included dystocia, postpartum hemorrhage, eclampsia, post-abortion care and caesarean section

### Factors associated with OOP payments

The percentage of women who paid for care differed significantly according to the type of health facility, health region, service**,** provider qualifications, and the women’s residence, age, education level, and occupation. Women paid OOP for 57.1% (*n* = 28) of cesarean sections, 47.6% (*n* = 10) of postpartum hemorrhage treatments, 39% (*n* = 23) of dystocia, 31.9% (*n* = 15) of dystocia with episiotomy, 36.8% (n = 2) of intrauterine manual vacuum aspirations, 28.6% (*n* = 7) of eclampsia treatments, 23.2% (*n* = 57) of normal deliveries and 23% (*n* = 32) of normal deliveries with episiotomy.

Table 3 presents the proportions of free and paid care services according to health facility and patients characteristics.

**Table 3:** Proportions of women who paid and who did not pay for services

Characteristics	Women who paid		Women who did not pay		*p*
	*n*	%	*n*	%	
Type of facility					< 0.001
Health center	83	24	263	76	
Medical center	11	27.5	29	72.5	
Hospital	80	39.8	121	60.2	
Region					< 0.001
Central	25	43.9	32	56.1	
Central-West	20	55.6	16	44.4	
Central-South	6	27.3	16	72.7	
Plateau Central	7	13.5	45	86.5	
Boucle du Mouhoun	25	29.8	59	70.2	
Cascades	12	42.9	16	57.1	
Hauts-Bassins	7	9.1	70	90.9	
Central-North	9	34.6	17	65.4	
Central-East	31	58.5	22	41.5	
Eastern	2	4.9	39	95.1	
Northern	13	36.1	23	63.9	
Sahel	2	6.3	30	93.7	
Southwest	15	34.9	28	65.1	
Type of service					< 0.001
Normal delivery	57	23.2	189	76.8	
Normal delivery with episiotomy	32	23.0	107	77.0	
Dystocia	23	39.0	36	61.0	
Dystocia with episiotomy	15	31.9	32	68.1	
Postpartum hemorrhage	10	47.6	11	52.4	
Post-abortion care	2	28.6	5	71.4	
Eclampsia	7	36.8	12	63.2	
Caesarean section	28	57.1	21	42.9	
Place of residence					< 0.001
Rural	111	24.9	335	75.1	
Urban	63	44.7	78	55.3	
Woman’s age (years)					0.009
14–19	36	22.1	127	77.9	
20–24	40	26.0	114	74.0	
25–29	42	34.7	79	65.3	
30–34	25	32.5	52	67.5	
35–48	31	43.1	41	56.9	
Woman’s education status					< 0.001
None	95	24.2	298	75.8	
Primary	41	36.3	72	63.7	
Secondary or higher	38	46.9	43	53.1	
Parity					0.301
Nulliparous	57	26.2	161	73.8	
Multiparous	98	31.1	217	68.9	
Grand multiparous	19	35.2	35	64.8	
Woman’s profession					< 0.001
Housewife	126	25.4	371	74.6	
Student	13	43.3	17	56.7	
Employed/Informal sector	35	58.3	25	41.7	
Provider’s qualification					< 0.001
Doctor	24	52.2	22	47.8	
Midwife	87	31.3	191	68.7	
Auxiliary midwife	38	22.4	132	77.6	
Nurses	16	20.8	61	79.2	
Surgical assistant	9	56.3	7	43.7	

Table 4 presents the adjusted results of the two-part model that included 576 observations in the first part and 163 in the second part. In the multiple logistic regression (first part), the category of service, health region and woman’s occupation were significantly associated with OOP payments. Hence, compared to housewives, women who were employed or working in the informal sector paid for services 2.34 times more often (95% CI = 1.14–4.81). Additionally, compared to the Central region, OOP payments were 3.77 times more common in the Central-East region (95% CI = 1.65–8.85), but were less common in the Hauts-Bassins (OR = 0.18; 95% CI = 0.08–0.43) and the Eastern (OR = 0.12; 95% CI = 0.02–0.55) regions. In the log-gamma regression (second part), the amount of OOP expenses decreased with age. However, the amount was higher in urban area and in hospitals. The woman’s occupation and the health region were also associated with the amount of OOP expenses. The predicted mean OOP expenses for the entire sample was US$1.44 (IC95%: 1.09–1.80). For a normal delivery, the average predicted OOP payment was US$0.90 (IC 95%: 0.41–1.40) versus US$1.77 (IC 95%: 1.24–2.30) for EmOC. In urban areas, patients paid an average of US$1.98 (IC 95%: 1.26–2.70) for a normally free service versus US$0.98 (IC 95%: 0.64–1.31) paid in rural areas.

**Table 4:** Two-part model for factors associated with OOP payments for deliveries and obstetrics emergencies (first part: logit model; second part: generalized linear model with log link and gamma distribution)

	First part: Odds ratios of positive OOP expenses (*n* = 576)			Second part: determinants of the amount of OOP expenses (*n* = 163)		
	OR	95% IC	*p*-value	coeff	95% IC	*p*-value
Woman’s age (years)						
14–19	1			0		
20–24	1.50	0.82–2.73	0.186	−0.552	−0.984, − 0.120	0.012
25–29	2.08	1.05–4.09	0.035	−0.596	−1.017, − 0.175	0.006
30–34	2.42	1.28–4.60	0.007	−0.054	− 0.603, 0.495	0.847
35–48	2.55	1.23–5.25	0.011	−0.762	−1.292, − 0.232	0.005
Type of facility						
Health center	1			0		
Medical center	0.62	0.19–2.00	0.423	0.466	−0.539, 1.471	0.363
Hospital	0.76	0.35–1.63	0.481	1.657	1.052, 2.262	< 0.001
Woman’s education						
None	1			0		
Primary	1.30	0.79–2.15	0.304	−0.128	−0.512, 0.255	0.512
Secondary or higher	1.75	0.82–3.78	0.150	0.467	0.080, 0.855	0.018
Place of residence						
Rural	1			0		
Urban	1.96	0.95–4.04	0.070	0.367	0.008, 0.727	0.045
Type of service						
Normal delivery	1			0		
EmOC	2.35	1.29–4.28	0.005	0.225	−0.327, 0.778	0.425
Woman’s occupation						
Housewife	1					
Student	1.36	0.56–3.30	0.501	−1.027	−1.541, −0.514	< 0.001
Employed/informal sector	2.34	1.14–4.81	0.021	0.019	−0.377, 0.415	0.926
Health region						
Central	1			0		
Central-West	2.32	0.96–5.62	0.062	−0.607	−1.148, − 0.065	0.028
Central-South	1.16	0.34–3.88	0.813	−0.097	−0.664, 0.469	0.737
Plateau central	0.38	0.13–1.09	0.073	0.247	−0.370, 0.864	0.433
Boucle du Mouhoun	1.01	0.33–3.05	0.990	−1.744	−2.187, − 1.302	< 0.001
Cascades	1.53	0.73–3.23	0.260	−0.757	−1.299, − 0.214	0.006
Hauts-Bassins	0.18	0.08–0.43	< 0.001	−0.525	−1.007, − 0.042	0.033
Central-North	1.75	0.42–7.35	0.446	−0.523	−1.069, 0.022	0.060
Central-East	3.77	1.65–8.85	0.002	−0.628	−1.120, − 0.136	0.012
Eastern	0.12	0.02–0.55	0.007	−1.798	−2.995, −0.600	0.003
Northern	1.26	0.53–3.02	0.605	−0.889	−1.374, − 0.404	< 0.001
Sahel	0.21	0.03–1.58	0.129	1.157	0.147, 2.166	0.025
Southwest	1.58	0.65–3.87	0.314	−0.773	−1.150, − 0.395	< 0.001

EmOC (emergency obstetric care) included postpartum hemorrhage, eclampsia, post-abortion care, dystocia and cesarean section

## Discussion

The results show that almost one-third of women made OOP payments for direct health care expenses incurred in deliveries or EmOC, even though this care should be completely fee-free. These results are consistent with the findings of previous studies that reported the persistence of OOP payments in Sub-Saharan Africa despite the adoption of cost-reduction policies [8, 10–14, 24]. However, OOP payments were less frequent in Burkina Faso than in other countries. For example, in a rural district in Tanzania, 62.5% of women reported in 2007 that they paid for delivery at a public health facility, despite the fact that this service was supposed to be free to patients [12], while in Ghana, 94.1% of women reported in 2016 that they paid for the direct health care expenses of vaginal delivery in a rural municipality [10].

Total OOP expenses varied greatly in the case of complications (coefficient of variation =147%) but not in the case of normal deliveries (CV = 163%). This variation was similar to that of drug expenses in both cases, which indicates that the variability of total OOP expenses is probably explained by the variability of drug expenses. It is not possible for the patient to predict the level of OOP expenditures when he goes to a health facility, thus this uncertainty can act as a barrier to health utilization despite the free care policy*.*

The purchase of drugs was also the main component of OOP payments that were made by 91.8% of women in Ghana in 2016, as reported by Dalinjong et al. [10]. According to the literature, the inadequate compensation offered by governments [9, 25, 26] and delays in reimbursement of covered services [10, 27–29] often cause disruptions in drug availability at health facility pharmacies. This situation forces patients to purchase drugs from private pharmacies, where they are usually more expensive. However, in Burkina Faso, health facilities cannot complain about inadequate compensation because the actual expenses of the services are paid by the Ministry of Health. In addition, there is no delay in reimbursement under the free care policy because the payments are made prospectively. These differences could explain why OOP payments are less frequent in Burkina Faso than in other countries in Sub-Saharan Africa.

The persistence of stock-outs at the health facility level indicates a problem in the supply and management of drugs and consumables. According to the Ministry of Health, nearly 60% of drug orders were completed by the company responsible for supplying public health facilities with essential generic drugs in 2017. The availability of drugs and medical consumables is essential to the success of the fee-exemption policy because drugs and consumables generally represent more than half of the direct health care expenses, as highlighted by Perkins et al. [14]. In countries such as Kenya and Tanzania, consultation fees account for two-thirds of the total medical expenses [12, 14]. In Burkina, the prices of consultations, drugs and consumables at public health facilities are capped by the government. Health facilities therefore do not have much flexibility in setting the prices.

The observed median OOP payment was US$1.77 (XOF1,050), with an IQR of 0.83–7.08, and the predicted mean was US$1.44 (IC 95%: 1.09–1.80). These amounts were far below the absolute monthly income threshold, estimated at US$21.58 (XOF12,794) per person in 2014 [30]. The amounts paid in Burkina Faso are relatively low compared to other countries, which suggests that the free care policy will have a positive impact on reducing the risk of household impoverishment. In Ghana, women paid an average of US$21.8 for vaginal deliveries, excluding hospitalization costs (min = US$1.5; max = US$444.48) in 2016 [10], while in Nigeria, they paid an average of US$3 for a normal delivery and US$93 for a cesarean section in 2010 [24].

At least one-tenth of the women paid for cleaning products (soap, bleach, detergent, etc.) to clean rooms and tables after the service, especially at the CSPS level. This practice is common in Burkina Faso that has already been reported by other authors [15]. These products are normally part of the state and local governments’ allocation to health facilities for their operations and should not be a cost borne by patients. However, the allotments for these needs are reported to be largely insufficient, and health facilities are often compelled to require each woman to pay for the products needed to clean the equipment used for her care.

The direct health care expenses of delivery found in this study were higher than those found in 2014, when health facilities were reimbursed and not prefunded. In 2014, the average direct health care expenses were US$5.36 (XOF3,180), US$22.44 (XOF13,305) and US$94.16 (XOF55,830) for a normal delivery, dystocia and cesarean section at the national level, respectively [31]. Although both studies used the same methodology, the two samples differed in several characteristics, and further analysis is required to find a potential link between the prefinancing of health facilities and the increase in expenditures.

In addition to stock-outs at health facility pharmacies, the desire for nonessential and nongeneric drugs may also require patients to shop at private pharmacies [10]. Several studies [32, 33] have shown that patients and health professionals believe that generic drugs are less effective than name-brand ones. Consequently, when patients have a certain purchasing power, some professionals prefer to prescribe branded drugs available only at private pharmacies. This practice is further facilitated by the presence of private pharmacies in the medical setting, which may partly explain why OOP payments are more prevalent among working women and the amounts are higher in urban areas and hospitals. However, the findings suggest that it may be cheaper for both households and the government if patients who do not need hospital level care use health centers or medical centers for service because both direct health expenses and OOP payments were lower in health centers and medical centers.

Delivery complications can lead to the prescription of specialty drugs purchased at private pharmacies and can explain the frequency of OOP payments in the case of EmOC. Thus, it may be beneficial for the free care policy to include commonly used specialty drugs in the hospital pharmacies in addition to essential and generic drugs. In Burkina, previous studies have shown that the success of district activities largely depends on the leadership of the district management team [34, 35]. Their role is particularly important in drug supply, and variations in the leadership of health officials could explain the differences in OOP payment frequency and OOP amounts by health region.

Previous studies [36, 37] have shown that there are inequalities in access to health care in Burkina Faso at the expense of rural areas, less educated patients and poor households. Our results (Table 4) showed that the risk of experiencing OOP payment did not differ according to the women’s education level and her place of residence. However, among those who paid, women with a secondary school level or more, and those living in urban areas paid more than their counterparts. In addition, those who were employed were more likely to experience OOP payment than students and housewives. Thus, the free care policy could contribute to reduce the inequalities in access to health care as reported by previous studies.

This study has some limitations. Interviews were conducted in health facilities, immediately after discharge. Therefore, direct health care expenses and OOP payments included only drugs purchased before discharge. No prescriptions that were pending (because the drug was not available during the visit or the patients did not have enough the money to purchase it) were included in the estimation of expenses. This exclusion could have led to an underestimation of direct health expenditures and OOP payments.

In addition, we could not determine all expenses by types of service and types of health facilities due to the small number of women who had some of the services. For the same reason, we were forced to group the eight services into two categories and this could have influenced the results of regression.

Finally, the study was conducted only six months after the introduction of the free care policy. Although some of the issues raised in this study may have been resolved over time, new concerns could also have arisen. Thus, there is a need for continuous monitoring and evaluation of such policies. Therefore, these results are useful for program managers as they document and gather evidence on factors that can hinder the effectiveness of this policy.

## Conclusion

The study showed that a significant proportion of women continue to pay for deliveries and EmOC, services that should be completely free of charge. These OOP payments were largely explained by drug and consumable stock-outs and drug prescriptions that were not available at the health facilities’ pharmacies. Improvement in facilities management and drug supply could thus improve the effectiveness of the free care policy.

## Abbreviations

CSPS: Centre de Santé et de Promotion Sociale
EmOC: Emergency obstetric care.
EmONC: Emergency obstetric and newborn care
MC: Medical center
MDG: Millennium Development Goals
NGO: Non-governmental organization
OOP: Out-of-pocket
UTH: University Teaching Hospital
